# Ending Restenosis: Inhibition of Vascular Smooth Muscle Cell Proliferation by cAMP

**DOI:** 10.3390/cells8111447

**Published:** 2019-11-16

**Authors:** Sarah A. Smith, Andrew C. Newby, Mark Bond

**Affiliations:** School of Translational Health Sciences, Faculty of Health Sciences, University of Bristol, Research Floor Level 7, Bristol Royal Infirmary, Bristol BS2 8HW, UK; S.Smith@bris.ac.uk (S.A.S.); A.Newby@bristol.ac.uk (A.C.N.)

**Keywords:** cAMP, VSMC, proliferation, cell cycle, actin, cytoskeleton, SRF, TEAD, CREB

## Abstract

Increased vascular smooth muscle cell (VSMC) proliferation contributes towards restenosis after angioplasty, vein graft intimal thickening and atherogenesis. The second messenger 3′ 5′ cyclic adenosine monophosphate (cAMP) plays an important role in maintaining VSMC quiescence in healthy vessels and repressing VSMC proliferation during resolution of vascular injury. Although the anti-mitogenic properties of cAMP in VSMC have been recognised for many years, it is only recently that we gained a detailed understanding of the underlying signalling mechanisms. Stimuli that elevate cAMP in VSMC inhibit G_1_-S phase cell cycle progression by inhibiting expression of cyclins and preventing S-Phase Kinase Associated Protein-2 (Skp2-mediated degradation of cyclin-dependent kinase inhibitors. Early studies implicated inhibition of MAPK signalling, although this does not fully explain the anti-mitogenic effects of cAMP. The cAMP effectors, Protein Kinase A (PKA) and Exchange Protein Activated by cAMP (EPAC) act together to inhibit VSMC proliferation by inducing Cyclic-AMP Response Element Binding protein (CREB) activity and inhibiting members of the RhoGTPases, which results in remodelling of the actin cytoskeleton. Cyclic-AMP induced actin remodelling controls proliferation by modulating the activity of Serum Response Factor (SRF) and TEA Domain Transcription Factors (TEAD), which regulate expression of genes required for proliferation. Here we review recent research characterising these mechanisms, highlighting novel drug targets that may allow the anti-mitogenic properties of cAMP to be harnessed therapeutically to limit restenosis.

## 1. Introduction

Vascular responses to injury include development of atherosclerotic plaques in response to cardiovascular risk factors, restenotic lesions after angioplasty, or intima formation during late vein graft failure, all of which are associated with increased rates of vascular smooth muscle cell (VSMC) proliferation. Since its discovery in 1958 by Earl Sutherland, the second messenger 3′ 5′ cyclic adenosine monophosphate (cAMP) is implicated in diverse physiological processes, including in the cardiovascular system, where physiological signals that elevate intracellular cAMP levels control vascular smooth muscle cell proliferation as well as smooth muscle cell migration and differentiation, vasodilatation, endothelial permeability, cardiac chronotropic and ionotropic responses, and cardiac hypertrophy. The ability of cAMP signalling to antagonise VSMC proliferation contributes towards normal vascular development and homeostasis and limits intimal thickening in response to injury, offering the opportunity for pharmacological interventions at multiple levels. However, it is only recently that we are beginning to fully understand the underlying molecular mechanisms and therefore develop more selective therapies. Here we review recent advances in cAMP signalling research that shed light on these important mechanisms.

## 2. Importance of Vascular Smooth Muscle Cell (VSMC) Proliferation

Vascular smooth muscle cells (VSMC) are highly specialised cells that reside in the media layer of blood vessels where their primary function is to contract and relax to regulate vessel tone and blood pressure. In healthy vessels, these contractile or differentiated VSMC express abundant contractile cytoskeletal proteins, but exhibit extremely low proliferation rates [1,2]. Nevertheless, VSMC retain the ability to dramatically increase their rate of proliferation in response to vascular injury, to repair damage to the vessel wall [3]. Defects in this repair capacity, due to replicative senescence or increase apoptosis, is associated with medial thinning and aneurysm formation [4,5] This capacity has no doubt evolved to compensate for traumatic injury, but its importance has recently become central in clinical cardiology and vascular surgery owing to its role in atherosclerosis and the two effective interventions developed to treat symptomatic atherosclerotic disease, angioplasty and venous by-pass graft surgery [6]. During atherosclerosis, medial VSMCs (or at least a small subpopulation of these) migrate to the developing lesion to generate a fibrous cap over lipid-rich lesions, which is believed to reduce the likelihood of thrombosis leading to myocardial, cerebral, renal and other tissue infarction [7,8]. The initial success of balloon angioplasty, often with metal stent implantation, in opening up vessels narrowed by atherosclerotic plaques is sometimes reversed owing to restenosis by expanded VSMCs and their associated connective tissue [9]. Likewise, vein by-pass grafts frequently fail thanks to growth of VSMC into a new occlusive intimal layer [10,11]. Proliferation of VSMC is believed to play a key role in both restenosis, where anti-proliferative agents reduce its frequency [12,13], and vein graft intima formation based on measurements of increased mitosis [14]. Hence targeting VSMC proliferation pharmacologically continues to be a clinically important goal.

The change in VSMC behaviour from contractile to proliferative is associated with a process termed phenotypic modulation [15], which inter alia is characterised by reduced expression of contractile proteins and increased expression of cell-cycle proteins that promote proliferation. Numerous signals are implicated in stimulating VSMC phenotypic modulation and increased VSMC proliferation, which are reviewed in detail elsewhere [16,17]. Briefly, this multi-step process includes inflammatory remodelling of the composition of the vascular extracellular matrix to one that promotes phenotypic modulation and is permissive to proliferation [18,19], and stimulation with VSMC mitogens such as platelet derived growth factors and fibroblast growth factors, which promote cell-cycle progression [20,21]. The primary purpose of VSMC proliferation is vascular repair after which it is essential that proliferation rates return to their normal low levels, an event that is associated with resolution of inflammation and re-establishment of normal endothelial function.

## 3. Anti-Mitogenic Effects of cAMP Signalling in VSMC

A large body of literature suggest that activation of cAMP signalling plays an important role in both the maintenance of VSMC quiescence in healthy vessels and the resolution of vascular repair mechanisms. The growth inhibitory properties of cAMP in cells of mesenchymal origin, which include VSMC, have been recognised for several decades [22], and this inhibitory effect is shared by many cells of other lineages. However, elevation of cAMP increases proliferation in embryonic stem cells [23], epithelial cells [24], endothelial cells [25,26], thyrocytes [27] and even VSMC under some conditions [28]. The mechanisms underlying this dichotomous behaviour have therefore sparked continuous interest, which is given greater impetus by the prospect it opens up for using cAMP elevation or some aspect of its downstream pathways to simultaneously decrease VSMC and increase EC proliferation, which should reduce the incidence of clinically evident restenosis without increasing the risk of endothelial denudation and hence late thrombosis.

One of the earliest reports documenting the anti-mitogenic effects of cAMP rather than cGMP in VSMC was in 1990, when Southgate et al. demonstrated that treatment of cultured rabbit aortic VSMC with the synthetic cAMP analogue 8-Bromoadenosine 3′,5′-cyclic monophosphate (8Br-cAMP) inhibited serum mitogen-induced DNA synthesis [29]. Since then many studies confirmed these early findings. For example, incubation of cultured VSMC with other synthetic cAMP analogues, including dibutyryl-cAMP [30,31,32], 8-Br-cAMP [33], 8-Chloro-cAMP [34] have similar growth inhibitory effects on VSMC in vitro. Furthermore, enhancing the rate of endogenous cAMP synthesis with the adenylyl cyclase activator, forskolin, or reducing the rate of cAMP degradation with phosphodiesterase inhibitors also strongly inhibits VSMC proliferation in vitro [25,31,32,35,36]. Consistent with the in vitro findings, treating vessels with these cAMP elevating agents in vivo results in inhibition of VSMC proliferation and intima formation in rodent models of vascular injury, importantly, without compromising endothelial integrity [32,33,34,37,38,39,40,41].

Synthesis of endogenous cAMP is balanced by cAMP hydrolysis, mediated by cyclic nucleotide phosphodiesterases (PDEs). In this way, PDE activity controls the amplitude, duration and compartmentalisation of cAMP signalling [42]. At least 60 different PDE isoenzymes encoded by 22 genes were described that together control intracellular levels of cAMP and cGMP. These are grouped into 11 families (PDE1-PDE11) based on their regulatory, kinetic and inhibitory properties [43,44,45], with different PDE displaying varying tissue specific expression and intracellular localisation patterns. Numerous studies demonstrated that PDE gene deletion or pharmacological PDE inhibition reduces VSMC proliferation in vitro and attenuates intima formation in vivo [35,36,38,46,47,48]. Depending on their phenotype, VSMC express PDE isoforms belonging to the PDE1, 3, 4 and 5 families [45]. Expression of *PDE1C* is low in quiescent contractile VSMC but elevated in synthetic VSMC in vitro and highly expressed in injury-induced neointimal VSMC of human coronary arteries. Importantly *PDE1C* gene deletion or pharmacological inhibition attenuated injury-induced VSMC proliferation and pathological vascular remodelling, consistent with a protective role of cyclic nucleotide signalling [49]. In synthetic VSMC, PDE1A is predominantly localised in the nucleus, compared to a cytoplasmic localisation in contractile VSMC. PDE1A activity has also been implicated promoting VSMC proliferation, in part through modulating levels of specific cell cycle regulatory proteins, including p27^Kip1^, Cyclin-D and p53 [50]. Although PDE1 isoenzymes preferentially hydrolyse cGMP, at least some of the growth inhibitory functions of these PDEs were attributed to elevated levels of cAMP [31,49,51]. PDE represent the major cAMP-hydrolysing PDE expressed in VSMC. Genetic deletion of PDE3A, but not PDE3B, inhibits mitogen VSMC proliferation, indicating a selective role of this PDE3 isoform in cell cycle regulation in these cells [52]. This suggests that novel therapies targeting specific PDE isoform might be effective in ameliorating excessive VSMC proliferation and intima formation.

In searching for physiological cAMP elevating agents, early studies identified the metabolite adenosine as a potent inhibitor of VSMC proliferation [53,54,55,56]. Treatment of VSMC in vitro with adenosine or stable adenosine analogues potently inhibited serum mitogen-induced proliferation. Adenosine is produced via catabolism of adenosine triphosphate (ATP), which is released from injured VSMC [57], and is rapidly metabolised by membrane bound enzymes, including ecto-5′-nucleotidase, to produce extracellular adenosine [57]. Extracellular adenosine mediates its effects of VSMC via a family of four G-protein coupled adenosine receptors that are classified by their ability to either activate or inhibit adenylyl cyclase. A1 and A3 adenosine receptors are G_0_ or G_i_ coupled and lead to increased intracellular Ca^2+^ ions or reductions in cAMP synthesis, respectively. By contrast, A2 receptor subtypes (A2A and A2B) are G_s_ coupled and their activation stimulates adenylyl cyclase activity and increases cAMP synthesis. Pharmacological studies using selective A2a or A2b receptor agonists and antagonists demonstrated that adenosine signalling through the A2B adenosine receptors inhibits of VSMC proliferation in vitro [25,54,55,56,58]. Moreover, A2B agonists reduce intima formation in rodent models of vascular injury in vivo [59]. These pharmacological effects may imply that activation of cAMP signalling in response to physiological stimuli such as adenosine that are present in the vessel wall play an important role in maintain VSMC in a quiescent contractile state in healthy vessels and limit excessive proliferation in response to vascular injury. Increased cAMP signalling is also likely to be important in promoting a return to quiescence as the healing process resolves. Consistent with this, inhibitory effects of A2BAR signalling on VSMC proliferation and intima formation were demonstrated using genetic studies where the A2BAR was knocked out [60] or silenced [61].

Prostacyclin production by the vascular endothelium represents another important physiological stimulus that represses VSMC proliferation by increasing cAMP levels in VSMC. Prostacyclin produced from a healthy endothelium by prostacyclin synthase binds to and activates the G_s_ coupled prostacyclin receptor (IP) on underlying VSMC to activate adenylyl cyclase and increase production of cAMP. Activation of IP receptors on VSMC using prostacyclin analogues potently inhibits VSMC proliferation [62,63,64], and intima formation in vivo [41,62,65,66], at least in part via adenylyl cyclase activation [67]. Consistent with these observations, genetic deletion of the prostacyclin receptor in mice is associated with exaggerated injury-induced vascular remodelling [68]. Endothelial dysfunction, which is often associated with a reduction in prostacyclin synthase expression [69], or injury-induced endothelial denudation dramatically reduces prostacyclin production within the vessel wall [70], thus removing this brake on VSMC proliferation. Conversely, regrowth of endothelium could help re-establish quiescence owing to restored prostacyclin production.

## 4. The Role of MAPK Signalling

Sustained activation of the p42/p44 MAPK kinase (ERK) pathway is a key step in mitogen-induced cell cycle progression [71]. Naturally, inhibition of MAPK signalling was one of the first mechanisms to be proposed to explain the anti-mitogenic effects of cAMP in cells of mesenchymal origin [72]. For example, early studies in fibroblasts and VSMC found that elevation of cAMP levels, either through addition of synthetic cAMP analogues, forskolin-activation of adenylyl cyclase, treatment with PDE inhibitors, or via stimulation of adenylyl cyclase with glucagon, cholera toxin or isoproterenol inhibited ERK activity induced by a range of mitogenic stimuli, including activation of receptor tyrosine kinases (RTK) (insulin, epidermal growth factor, platelet-derived growth factor [PDGF] isoforms), activation of GPCRs (lysophosphatidic acid (LPA)) or phorbol ester-mediated activation of protein kinase C (PKC) [73,74]. However, inhibition of p42/p44 ERK activation by cAMP is transient [75,76] and only partially reduces the sustained phase of ERK activation induced by some (e.g., PDGF_BB_) but not all (e.g., bFGF) mitogens [74,77]. Hence, cAMP induces a temporal shift (delay) in mitogen stimulated MAPK signalling, although this alone could not account for the growth inhibitory action of cAMP [75,76]. Furthermore, several lines of evidence support the conclusion that the anti-mitogenic effects of cAMP can be dissociated from inhibition of ERKs [77]. For example, cAMP elevating agents retain the ability to strongly inhibit proliferation even when they are added several hours after growth factor induced ERK activity has peaked and returned to basal levels [77], whereas delayed addition of PD98059 an inhibitor of MEK, the kinase upstream of ERK, had no effect of proliferation. Finally, cAMP signalling can inhibit proliferation in cells in which MEK activity has already been inhibited with PD98059 [77]. Taken together, these studies suggest that the transient effects of cAMP on MAPK signalling at best only partially explains the anti-mitogenic properties of cAMP and implicate additional mechanisms.

## 5. Cell Cycle Inhibition by cAMP

**Figure 1:**
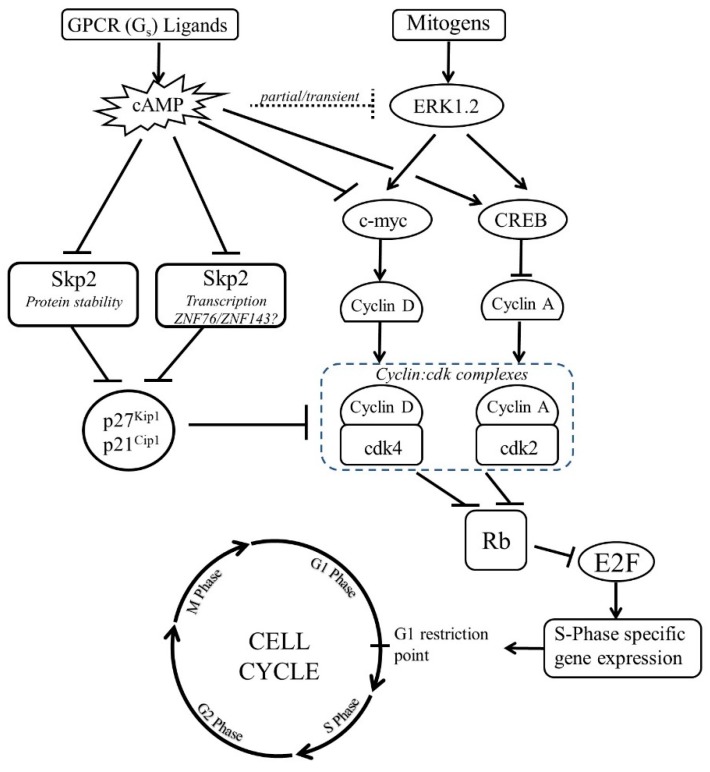
Cell cycle regulation by cAMP in VSMC. In VSMC, elevated cAMP inhibits S-phase entry. S-phase entry is controlled by retinoblastoma protein (Rb, which represses E2F-dependent gene expression. Rb inactivation requires phosphorylation by cdk enzymes, which are dependent on binding to cyclin proteins for catalytic activity. Expression of cyclin-A and -D is repressed by cAMP, via reductions in c-myc expression and activation of CREB respectively. Activity of cyclin:cdk complexes is inhibited by CDKIs, including p27^Kip1^ and p21^Cip1^. Levels of p27^Kip1^ and p21^Cip1^ are reduced in late G_1_ phase via Skp2-mediated ubiquitination and proteasomal degradation. Skp2 levels normally increase in late G_1_ to remove this brake on cdk activity. However, cAMP represses induction of Skp2, which increases p27^Kip1^ and p21^Cip1^ levels. This reduction in cyclin expression and increase in CDKI levels, prevents phosphorylation of Rb and entry into S-phase.

Numerous studies show that cAMP inhibits VSMC proliferation by blocking progress from G_1_ into S-phase of the cell cycle, where new DNA is synthesised [29,31,78,79,80]. For example, elevated cAMP reduces incorporation of thymidine analogues, such as tritiated thymidine or bromodeoxyuridine, indicating reduced DNA synthesis. The observation that cAMP retains the ability to inhibit proliferation even when addition of cAMP elevating agents is delayed by up to 12 hours after mitogen stimulation [77,81] implies its growth inhibitory properties are mediated, at least in part, by targeting events in the mid to late G_1_ phase of the cell cycle. Progression from late-G_1_ into S-phase is controlled at the G_1_-restriction point, which is centred on the retinoblastoma protein (Rb), which sequesters the E2F transcription factors that are needed for S-phase specific gene expression [82]. Hyper-phosphorylation of the Rb protein by cyclin-dependent kinases (CDK), particularly CDK4 and CDK2, represents the core regulatory mechanism controlling Rb inactivation. CDK activity is dependent on binding to their regulatory partners, the cyclins, levels of which fluctuate in synchrony with the cell cycle, owing to phases of synthesis and degradation. D-type cyclins, such as Cyclin-D_1_ are the first cyclins to be synthesised following mitogen stimulation [71]. Cyclin-D:cdk4 complexes phosphorylate Rb, triggering E2F activation. Targets of E2F include Cyclin-E, Cyclin-A and many other S-phase specific genes. Thus, cyclin proteins control the timing of CDK activity (provided there is not an excess of inhibitors present) and therefore play an important role in regulating S-phase entry. Several studies demonstrated inhibition of cyclin protein production in response to cAMP elevating stimuli (see Figure 1). For example, stimulation of VSMC with the cAMP analogues, 8-Bromo-cAMP or dibutyryl-cAMP, reduced mitogen-induced cyclin-D_1_ expression and inhibited activity of its catalytic partner, Cdk4 [78,79,83,84]. Similar effects have been reported for forskolin, which decreases PDGF_BB_ or serum mitogen-induced expression of cyclin-D_1_ protein [77,78]. Inhibition of cyclin-D_1_ levels likely occurs as a result of transcriptional inhibition, since both mRNA levels and cyclin-D_1_ promoter activity are reduced by cAMP elevating stimuli [78,85]. This is mediated, at least in part, by repression of the transcription factor, c-myc [77,86], an important inducer of cyclin-D_1_ gene expression [87]. Importantly, forced constitutive expression of either c-myc or cyclin-D_1_ significantly reduces the growth inhibitory actions of cAMP, confirming the importance of this mechanism [88,89]. Cyclin-A expression has also been reported to be repressed by cAMP elevating stimuli [90]. Kamiya et al. [90] reported that cAMP analogues or forskolin inhibit cyclin-A gene transcription via a mechanism that is dependent on serine-133 phosphorylation of the cyclic-AMP response element binding protein (CREB) transcription factor, a post-translational modification typically associated with CREB activation. However, Kothapalli et al. [91] reported that the prostacyclin analogue, cicaprost, represses cyclin-A expression by reducing CREB binding to the cyclic-AMP response elements (CRE) in the cyclin-A promoter. Interestingly, treatment of VSMC with 8-Bromo-cAMP [84] or forskolin [78] did not inhibit expression of Cyclin-E, even though activity of the cyclin-E:cdk2 complex was inhibited. This may reflect the fact that cyclin-E can be expressed throughout the G_1_ phase and that activity of the cyclin-E:cdk2 complex is typically regulated by levels of the cdk-inhibitors (CKIs), p21^Cip1^ and p27^Kip1^ [84,92]. These observations focus attention on the role of CKIs in the anti-mitogenic effects of cAMP.

In the early G_1_ phase, cdk activity is subject to negative regulation by the Cip/Kip (p21^Cip1^, p27^Kip1^ and p57^Kip2^) and Ink (p15^INK4a^, p16^INK4b^, p18^INK4c^ and p19^INK4d^) families of cyclin-dependent kinase inhibitors (CKIs) [93]. However, levels of p21^Cip1^ and p27^Kip1^ are typically down regulated in response to mitogen stimulation, effectively removing the brake on cdk activity. Numerous studies have demonstrated that cAMP elevating stimuli increase the levels of p21^Cip1^ and p27^Kip1^ in VSMC. For example, treatment of cultured VSMC with forskolin or cAMP analogues prevents mitogen-induced down-regulation of p27^kip1^ [32,77,84,94] and p21^Cip1^ [95]. Similar increases in p27^Kip1^ and p21^Cip1^ are also observed in VSMC stimulated with GPCR agonists that elevate endogenous cAMP levels. For example, the prostacyclin mimetics Beraprost [41,96] or Cicaprost [97] significantly increase levels of p27^Kip1^ in VSMC. Unlike the cyclins, levels of CDKI mRNA remain relatively constant throughout the cell cycle. Instead, CDKI protein levels are typically controlled by rapid ubiquitin-dependent proteasomal-mediated degradation in response to mitogen stimulation [98,99]. This suggests that cAMP may inhibit proliferation by preventing the proteasomal degradation of CDKIs, effectively restring the brake on proliferation. In 1999, the discovery of Skp2, an F-box protein component of the SCF^skp2^ ubiquitin ligase, responsible for p27^Kip1^ polyubiquitylation and degradation [100] ultimately provided a mechanistic link between cAMP and CDKI levels. Skp2 protein levels increase in late-G1, triggering p27^Kip1^ and p21^Cip1^ degradation and cdk activation [100]. However, elevated cAMP completely blocks mitogen-induction of Skp2 [32,79], thereby explaining the effects of cAMP on CDKI levels. Moreover, forced expression of Skp2, rescues p27^Kip1 and^ p21^Cip1^ down-regulation and Rb-hyper-phosphorylation in forskolin stimulated VSMC [79,101]. Skp2 overexpression also partially restores VSMC proliferation, suggesting that cAMP-induced down-regulation of Skp2 is a key mechanism contributing to cell cycle arrest in VSMC [79]. Exactly how cAMP controls Skp2 levels is unclear. Stimulation of VSMC with dibutyryl-cAMP reduces Skp2 protein levels at least in part by enhancing Skp2 protein turnover [32]. However, cAMP elevating stimuli also reduce Skp2 mRNA levels and activity of the Skp2 promoter, implicating transcriptional repression [32]. Skp2 mRNA levels are low in differentiated VSMC that have very low rates of proliferation but are elevated in phenotypical modulated VSMC that proliferate rapidly in response to mitogens [79]. Lack of Skp2 gene expression in differentiated VSMC explains why these cells are unable to down-regulate p27^Kip1^ and proliferate, even when exposed to strong mitogenic stimulation [102]. Furthermore, transcriptional up-regulation of Skp2 during VSMC phenotypic modulation effectively primes these cells to be able to respond to mitogenic stimulation. Transcription of the Skp2 genes in proliferating VSMC is controlled by two closely related transcription factors, ZNF143 and ZNF76 [103]. However, it is not known if these are the targets for cAMP repression of Skp2 expression.

## 6. The Role of cAMP Effectors

Several cAMP sensitive proteins are described, including protein kinase A (PKA), exchange protein activated by cAMP -1 and -2 (EPAC1/2), cyclic nucleotide gated ion channels (CNG) and the Popeye domain containing (Popdc) family of proteins.

Protein Kinase A, first discovered in 1968 [104], is one of the best studied members of the serine/threonine protein kinases. In an inactive state, PKA consists of two catalytic subunits and two regulatory subunits bound together as a tetrameric holoenzyme. Each regulatory subunit contains two cAMP binding sites. The classical model of PKA activation involves binding of cAMP to the regulatory subunits, causing a change in conformation that results in dissociation and activation of the catalytic subunits that then phosphorylate their substrates [105]. However, some reports suggest that cAMP can activate PKA without catalytic subunit release [31,106,107,108] and that intact and active holoenzymes exist within the cytoplasm in the presence of cAMP. However, other studies suggest that a large molar excess of regulatory subunits over catalytic subunits is important for reducing catalytic subunit diffusion and increasing catalytic subunit recapture rate [109]. There are four PKA regulatory subunits (RIα, RIIα, RIβ and RIIβ) and three catalytic subunits (Cα, Cβ and Cγ). PKA regulatory subunits combine with different catalytic subunits to form various PKA isoforms that display different biochemical properties [110,111]. The four PKA regulatory subunits are structurally and functionally diverse; they display cell type specific expression patterns and different subcellular localisations [109,112,113,114], allowing for fine tuning of PKA activation and cellular responses. PKA holoenzymes interact with members of the A-kinase anchoring proteins (AKAPs) that tether PKA isoforms to distinct cellular locations in the vicinity of specific substrates. Studies using either pharmacological or peptide-based PKA inhibitors support a requirement for PKA activity in mediating the anti-mitogenic effects of cAMP [33,115,116,117,118,119]. Consistent with this, PKA inhibition reverses the inhibitory effects of cAMP elevating agents on neointima formation in a rat model of vascular injury [33]. It is important to note, however, that PKA activation has also been implicated in pro-mitogenic signalling in VMSC [28,120]. For example, PKA inhibition reduces VSMC proliferation induced by purinergic stimulation [28]. This likely reflects the importance of temporal regulation of PKA activation in determining cellular responses to cAMP elevating stimuli. Stimulation of purinergic receptors on VSMC results in a transient activation of PKA, which is essential for the proliferative response [28], which contrasts with the sustained activation of PKA induced by forskolin or prostacyclin analogues that inhibit VSMC proliferation [28]. However, sustained activation of PKA alone using a PKA selective cAMP analogue (6-BNZ-cAMP) does not significantly inhibit vascular [79] or airway smooth muscle cell proliferation [121], implying involvement of additional cAMP sensors in the anti-mitogenic response.

PKA has a wide repertoire of substrates that contain the consensus PKA phosphorylation sites based on Arg-Arg-X-Ser/Thr, Arg/Lys-C-C-Ser/Thr and Arg/Lys-X-Ser/Thr motifs [122]. Vasodilator-stimulated phosphoprotein (VASP) is a well characterised PKA substrate that is implicated in growth regulation. VASP is preferentially phosphorylated by PKG at serine239, whereas serine157 is the preferred phosphorylation site for PKA. In VSMC, VASP is associated with actin filaments, focal adhesions and cell-cell contacts, where is plays a role in regulating cell motility and proliferation [123]. Precisely how VASP phosphorylation at serine157 regulates VSMC proliferation is not clear. This residue can be phosphorylated in response to mitogen and phorbol ester stimulation, as well as in response to cAMP-mediated activation of PKA, in response to forskolin, cAMP analogues, ATP, endothelin or isoproteranol [28,79,124]. Ectopic expression of VASP containing a mutated PKA phosphorylation site (S157A) results in impaired mitogen stimulated proliferation, implying that phosphorylation at this site is required for VSMC proliferation [123]. This suggests that PKA-mediated phosphorylation of VASP may contribute towards the pro-mitogenic effects of transient PKA activation that occurs in response to purinergic stimulation [28] and not the anti-mitogenic effects of sustained PKA activation. Although PKA is generally accepted to represent the major serine/threonine kinase activated by cAMP, several studies have suggested that some of the biological effects of cAMP may, at least in part, be mediated via activation of Protein Kinase G (PKG) [32,125,126], which is classically activated by the second messenger cyclic guanosine monophosphate (cGMP). The cyclic nucleotide binding domain of PKG binds cGMP with higher affinity than cAMP. However, the intracellular concentrations of cAMP are typically higher than those of cGMP, suggesting that the selectivity of PKG for cGMP compared to cAMP is not controlled uniquely through affinities and cAMP can act as a partial PKG agonist [127].

Clearly, not all the biological effects of cAMP are mediated by PKA and PKG. The exchange proteins activated by cAMP (EPACs) were discovered while studying the mechanism of cAMP-mediated activation of Rap1 GTPase, which was insensitive to PKA inhibition [128]. A second study independently identified EPACs by screening for brain enriched genes that containing cAMP binding motifs [129]. Two EPAC isoforms (EPAC1 and EPAC2) were described [129], both of which contain an N-terminal cAMP binding regulatory region and a C-terminal catalytic region responsible for guanine nucleotide exchange activity. EPAC1 has a single cyclic nucleotide binding domain while EPAC2 has two. Deletion of the N-terminal CNB domain(s) results in a fully active catalytic domain, suggesting a mechanism whereby the CNB auto-inhibits the catalytic domain [130]. A conformational change in the CNB induced by cAMP binding relieves the inhibition of the catalytic domain. Numerous studies have implicated EPAC-dependent signalling, either in parallel or independently of PKA, in the regulation of cell proliferation [40,79,121,131,132,133,134,135,136,137,138,139,140,141,142,143,144,145,146,147,148]. The effects of EPAC activation on cell proliferation is cell type specific, with growth inhibitory effects in VSMCs and some other cell types [79,131,134,149,150] and growth promoting effects in others, including endothelial cells [133,146,151]. Changes in the relative levels of cAMP sensors during vascular remodelling may contribute towards different functional responses. EPAC1 expression is increased following vascular injury in vivo, concomitantly with a decrease in PKA regulatory and catalytic subunit expression, implying a bias towards EPAC1 signalling following tissue injury that may be involved in promoting vascular repair [152]. However, its precise role in vascular remodelling may be complex. In isolated VSMC, selective EPAC activation alone using 8-(4-chloro-phenylthio)-2′-*O*-methyladenosine-3′-5′-cyclic monophosphate (8-pCPT-2′-O-Me-cAMP) or over expression of EPAC1 does not affect proliferation [79,152,153]. However, EPAC activation synergistically enhances the anti-mitogenic effects of selective PKA activation in VSMC in vitro [79]. Pharmacological EPAC activation reduces intima formation in an ex vivo human saphenous vein organ culture model [148] and EPAC1 gene deletion in mice promotes VSMC proliferation in vitro [154], consistent with an anti-mitogenic role of EPAC1 signalling. On the other hand, stimulation of ex vivo arterial organ cultures with 8-pCPT-2′-*O*-Me-cAMP or over expression of EPAC1 increases intimal thickening [152], whereas deletion of the EPAC1 gene in mice reduces intima formation and VSMC proliferation in wire-injured femoral arteries [154,155]. Moreover, pharmacological inhibition of EPAC with ESI-09 recapitulates the EPAC1 null phenotype and is sufficient to reduce intima formation [155]. These paradoxical effects of EPAC signalling on VSMC proliferation in vitro and intima formation in vivo may be at least partly explained by the ability of EPAC1 to enhance VSMC migration [152,153]. A further complication is that EPAC forms functionally compartmentalised signalling complexes with phosphodiesterase enzymes [121,122], which may be disrupted by EPAC1 gene deletion or silencing. Hence, data from these experiments needs to be interpreted cautiously.

Several mechanisms have been suggested to explain the positive and negative effects of EPAC1 on cell proliferation. Elevation of cAMP typically leads to the EPAC-dependent activation of Rap1, which has also been linked to both stimulatory and inhibitory effects on cell proliferation, depending on the cellular context. The Rap1b gene was originally identified by its ability to revert Ras-transformation [156]. Although the effector domains of Rap1b and Ras are almost identical, Rap1b is unable to activate Ras effector proteins, despite physically interacting with them [157,158,159,160,161,162]. In this model, Rap1b inhibits mitogenic signalling by antagonising Ras function. However, Rap1 has also been shown to have a mitogenic function in some cell types, typically those where cAMP is mitogenic [161,163,164].

Interestingly, EPAC-mediated activation of Rap1 does not appear to be involved in the anti-mitogenic effects of cAMP in VSMC. Evidence includes that selective activation of EPAC in VSMC does not inhibit proliferation despite rapid and robust Rap1 activation [79]. Furthermore, constitutively active mutants of Rap1 actually promote proliferation in VSMC [165] and inhibition of Rap1 activity by overexpression of Rap1GAP does not reverse cAMP-induced growth arrest [79]. This implicates other EPAC effectors, the identity of which are currently unknown. One possibility is that EPAC activation acts as a modulator of PKA signalling. For example, microarray analysis of VSMC stimulated with a selective EPAC agonist (8-pCPT-2′-*O*-Me-cAMP) for 8 h did not reveal any significantly regulated genes [139]. However, EPAC activation modulated expression of PKA-sensitive genes such as Egr1 that plays a central role in the regulation of VSMC proliferation. Hence, EPAC signals converge with PKA signals to modulate expression of genes involved in cell proliferation.

Cyclic nucleotide gated ion channels are nonselective cation channels that open in response to direct binding of cAMP and cGMP. They are typically expressed in cone photoreceptor cells and olfactory sensory neurons [166], although they have also been detected in VSMC [167,168]. However, there is currently no evidence that CNG channels are involved in mediating the effects on cAMP on cell proliferation.

The Popdc proteins (Popdc1, Popdc2, Popdc3) are a family of integral membrane proteins that contain an extracellular N-terminal domain, three transmembrane domains and a cytosolic Popeye domain, which functions as a high affinity cAMP binding site [169]. Some downstream effects of binding of cAMP to Popdc proteins were described, including a dissociation of Popdc proteins from the cardiac two-pore potassium channel, TREK1, resulting in a reduction of the TREK-1 current [169]. There is currently no direct evidence that Popdc proteins play a role in the regulation of cell proliferation in response to cAMP elevating stimuli. However, Popdc proteins can interact with several proteins that are involved in mitogenic signalling. For example, Popdc1 physically interacts with guanine exchange factor T (GEFT), a guanine exchange factor (GEF) for Rho GTPases, including RhoA that play an important role in regulating actin polymerisation and mitogenic signalling [170]. In NIH3T3 fibroblasts, overexpression of GEFT promoted cell proliferation and migration [171]. Popdc proteins have also been implicated in modulation of the Wnt signalling pathway, which promotes transcription of genes required for VSMC proliferation [172,173,174,175]. Deletion of the Popdc1 gene results in increased Wnt activity, increased levels of β-catenin and enhanced expression of Wnt target genes, implying that Popdc1 represses Wnt/β-catenin signalling [176]. Popdc1 knockout cells also display increased c-myc levels, due to the loss of Popdc1 mediated destruction of c-myc protein [176].

## 7. The Role of Actin Cytoskeleton Remodelling

**Figure 2:**
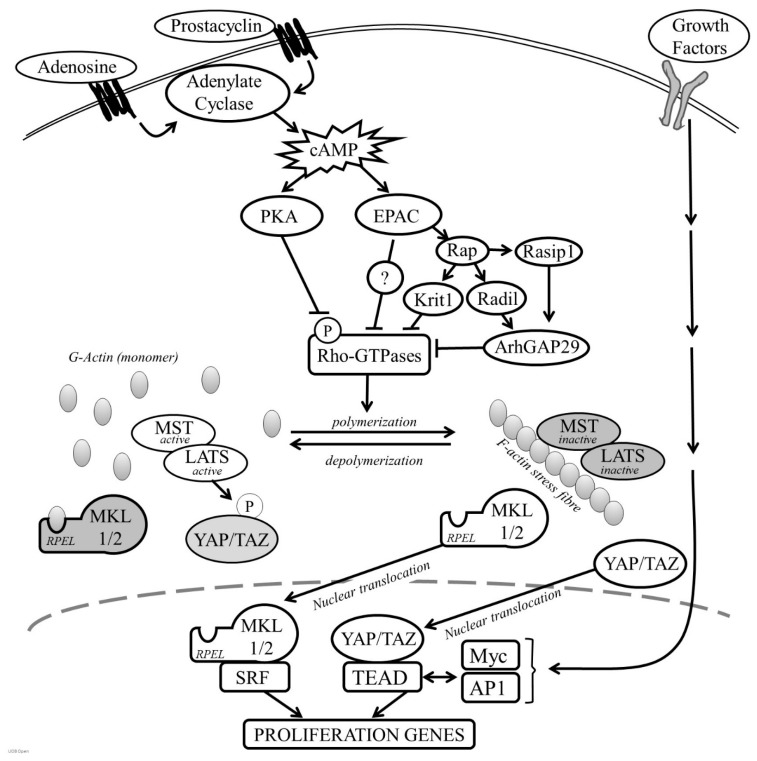
Role of actin remodelling in cAMP-induced growth arrest. In VSMC, cAMP activates Protein Kinase A (PKA) and Exchange Protein Activated by cAMP (EPAC), which synergise to inhibit RhoGTPase activity. EPAC1 activation can contribute towards RhoGTPase inhibition via Rap1 mediated activation of Krit1, Radil and Rasip1. However, some studies suggest that EPAC1 inhibits actin polymerisation and proliferation via a Rap1-independet mechanism. Under low cAMP conditions, the F:G actin ratio is high, allowing Megakaryocytic Acute Leukemia Protein (MKL)-1 and -2 to translocate to the nucleus where they activate serum response factor (SRF)-dependent gene expression. Actin polymerisation also inhibits the Hippo kinases Mammalian Sterile20-like (MST) and Large Tumor Suppressor Kinase (LATS), allowing YAP and TAZ to translocate to the nucleus where they activate TEA Domain (TEAD) transcription factor dependent gene expression. Reduced actin polymerisation in response to elevated cAMP causes an increase in actin monomer levels and activation of MST and LATS. LATS-mediated phosphorylation of Yes-Associated Protein (YAP) and Transcriptional Co-Activator With PDZ-Binding Motif (TAZ) triggers their nuclear export. Actin monomer binds to Megakaryocytic Acute Leukemia Protein (MKL) -1 and -2 via their N-terminal RPEL domains, triggering their nuclear export. This results in a reduction in the expression of serum response factor (SRF) and TEAD-dependent gene expression required for cell proliferation.

Cyclic-AMP induced growth arrest in VSMC is associated with a profound but reversible change in cell morphology resulting from reorganisation of the actin cytoskeleton [32,79,94,177,178,179,180,181]. Stimulation of VSMC with forskolin, cAMP analogues or activation of the prostacyclin, adenosine A2B receptors or β-adrenergic receptors, results in a rapid acquisition of a ‘stellate’ morphology, characterised by a condensation of the cell body and formation of long branching processes. These changes were reported after as little at 10–15 min following stimulation, suggesting that they are an early event following cAMP elevation and occur independently of changes in gene expression [25,177]. Instead, these morphological changes are accompanied by loss of F-actin stress fibres and a decrease in the F:G actin ratio, indicating that cAMP inhibits actin polymerisation in VSMC [25]. Consistent with this, cAMP elevating stimuli inhibit the activity of members of the Rho GTPases (see Figure 2), including RhoA and Rac1 that play a central role in controlling actin polymerisation [94,177]. RhoGTPase inhibition appears to be a critical step since forced expression of constitutively active mutants of RhoA or Rac1 reverse cAMP-induced morphological changes, F-actin disassembly and inhibition of proliferation [94,182]. PKA can directly phosphorylate RhoA on Serine-188, a modification that terminates RhoA signalling by triggering translocation from the membrane to the cytosol, without affecting its ability to hydrolyse GTP [182]. However, cAMP elevating stimuli can also reduce levels of GTP bound RhoA, implying the existence of multiple mechanisms that control RhoGTPase function in response to cAMP. PKA can also directly phosphorylate G-actin, which reduces the rate of actin polymerisation [183]. Importantly, selective PKA activation with 6-BNZ-cAMP does not induce a strong stellate morphology or F-actin disassembly, implying that PKA-mediated phosphorylation of RhoA alone is insufficient [79]. Instead, simultaneous PKA and EPAC activation is necessary for these morphological changes and actin cytoskeleton remodelling. PKA and EPAC synergise to inhibit the activity of the RhoGTPase Rac1 [139], mirroring their synergistic action on growth arrest, but how EPAC cooperates with PKA to repress RhoGTPase activity is unclear. EPAC activity has also been linked to suppression of RhoA activity via the Rap1 effector proteins KRIT-1/CCM [184], Radil and Rasip1. Radil and Raspi1 inhibit RhoA activity via the RhoGAP, ArhGAP29 [185]. Taken together, these observations suggest that PKA and EPAC signalling pathways converge at the level of Rho GTPase activity to modulate actin cytoskeleton dynamics.

Cell shape and actin cytoskeleton integrity is a critical regulator of cell proliferation in adhesion dependent cells. Studies using actin-binding drugs such as cytochalasin-D have revealed that actin cytoskeleton integrity is essential for cells to progress past the G_1_-restriction point [186,187]. Early work focussed on impacts to the MAPK pathway [188,189], but using delayed addition of either cytochalasin-D or the MEK inhibitor PD98059 established that the ability of a properly organised actin cytoskeleton to promote cell cycle progression is not mediated simply via MAPK signalling. MAPK signalling is only essential in the early G_1_ phase of the cell cycle whereas cytoskeleton integrity is essential throughout most of the G_1_ phase, right up until the restriction point [187]. Clearly the effects of the actin cytoskeleton on proliferation can be dissociated from its effects on MAPK activity, much in the same way that the anti-mitogenic effects of cAMP signalling can be dissociated from effects on MAPK signalling [77], implying that loss of cytoskeletal integrity mediates the antiproliferative effects of cAMP.

Several lines of evidence confirm that RhoGTPase inhibition and a reduction in actin polymerisation underlie the anti-mitogenic effects of cAMP in VSMC. For example, forced expression of constitutively active mutants of RhoA (RhoAG14V) or Rac1 (Rac1G12V) reverse cAMP-induced morphological changes, restore actin polymerisation and rescue cell proliferation [94,177]. Genetic or pharmacological inhibition of RhoGTPase activity or direct disruption of actin polymerisation with cytochalasin-D, mimic cAMP-induced morphologic changes and inhibition of VSMC proliferation [94,139]. Cytochalasin-D or elevated cAMP also inhibit expression of actin-sensitive genes required for proliferation, including CCN1, CTGF and EGR1 [139,190]. Promoting actin polymerisation either with active RhoGTPase mutants or the actin-binding drug jasplakinolide rescues expression of these genes [139,190,191]. Finally, cAMP signalling is typically anti-mitogenic in cells where cAMP inhibits actin polymerisation e.g., VSMC [79,94,177] and fibroblasts [192], whereas, in endothelial cells, cAMP promotes formation of cortical actin stress fibres and does not inhibit proliferation [25,193,194].

The mechanisms linking actin cytoskeleton remodelling to changes in gene expression allow cells to sense and respond to various microenvironmental signals, including those that elevate cAMP. An important insight into these mechanisms came from studies analysing the regulation of the *c-fos* gene, which is controlled by binding of serum response factor (SRF) to a serum response element (SRE) in the *c-fos* promoter. Mitogen activation of MAPK signalling stimulates the *c-fos* promoter by inducing ternary complex factor (TCF) binding to SRF. However, promoter mutants incapable of binding TCFs remain responsive to signals that can activate Rho GTPase activity and induce cytoskeleton rearrangement [195,196]. Subsequent studies established that SRF activity is also sensitive to actin cytoskeleton dynamics, being inhibited by increases in monomeric actin [197]. The PKA substrate VASP is implicated in the regulation of actin polymerisation and VASP expression promotes SRF activity via a RhoA-dependent mechanism, suggesting that PKA-dependent phosphorylation of VASP is involved in the cAMP-mediated regulation of SRF [28,124,198,199,200]. Regulation of SRF activity in response to actin remodelling is mediated by the actin-binding SRF co-factors, Myocardin Related Transcription Factor A (MRTF-A/MKL1) and Myocardin Related Transcription Factor B MRTF-B/MKL2), which associate with G-actin via their N-terminal RPEL motifs (see Figure 2) [201]. Signals that activate Rho GTPases and promote actin polymerisation liberate MKL1 and MKL2 to translocate into the nucleus where they bind and activate SRF. Conversely, signals that inhibit actin polymerisation, enhance association of actin monomer with the MKL RPEL motifs, triggering their nuclear export and reduce expression of SRF-dependent immediate early genes. Consistent with this model, cAMP-induced actin remodelling in VSMC induces MKL1/2 nuclear export and inhibits MKL-SRF-dependent gene expression [25]. This mechanism accounts, at least in part, for the anti-mitotic effects of cAMP in VSMC, given that pharmacological or siRNA-mediated inhibition of MKL1 and -2 reduces VSMC proliferation in vitro [25] and genetic deletion of MKL1 reduces neointima formation in wire-injured femoral arteries in mice [202]. Interestingly, in endothelial cells, MKL1 remains constitutively nuclear, even after cAMP stimulation, a difference that likely reflects the maintenance of actin stress fibres and low levels of actin monomer in endothelial cells and probably explains the divergent effects of cAMP signalling on proliferation in these two cell types.

The transcriptional coactivators Yes-associated protein (YAP) and transcriptional co-activator with PDZ-binding motif (TAZ) also play an important role in linking actin cytoskeleton dynamics to gene expression and changes in cell behaviour [203]. In the nucleus, YAP and TAZ interact with members of the TEA domain (TEAD) transcription factors and several other components of the transcriptional machinery, to regulate gene expression [204,205] and promote cell proliferation [206]. The functions of YAP and TAZ are negatively regulated by the Hippo pathway kinases MST and LATS [207,208]. LATS-mediated phosphorylation inactivates YAP and TAZ by triggering their nuclear exclusion and proteasomal degradation, leading to a reduction in TEAD-dependent gene expression [209,210]. This direct regulation of YAP and TAZ by LATS kinase is termed ‘canonical signalling’ in contrast to non-canonical LATS-independent mechanisms [211]. Recent studies showed that YAP and TAZ activity is sensitive to a wide range of environmental signals that modulate actin cytoskeleton dynamics and actin cytoskeleton tension, including extracellular matrix stiffness, adhesion, cell-cell contact, cell shape and G-protein-coupled receptor activation [212]. Actin polymerisation promotes YAP and TAZ nuclear localisation and TEAD activation either via inactivation of the Hippo kinases MST and LATS or via non-canonical pathways [211]. This results in a reduction in YAP and TAZ phosphorylation, permitting their nuclear accumulation where they drive cell proliferation [203,213,214]. Since cAMP elevating stimuli inhibit RhoGTPase activity and actin polymerisation [94,177], they rapidly induce YAP and TAZ phosphorylation, nuclear exclusion and reduced TEAD activity [94,191]. Moreover, silencing of YAP and TAZ inhibits VSMC proliferation in vitro and silencing or VSMC-specific deletion of YAP reduces neointimal formation in vivo [191,215,216], demonstrating their importance in VSMC proliferation. Constitutively active mutants of YAP and TAZ completely reverse cAMP-induced growth arrest, demonstrating that their inhibition contributes towards the anti-mitogenic effects of cAMP [191]. Analysis of the YAP induced transcriptome indicates that YAP-dependent activation of TEAD promotes cell proliferation by enhancing the expression of a wide range of proliferation associated genes [217], including CCN1 and CTGF that were implicated in VSMC proliferation [190,218] as well as key cell cycle regulators, such as Cyclin-D [215]. Interestingly, CCN1 and CTGF are also subject to regulation by the MKL-SRF axis [25], showing that genes required for efficient VSMC proliferation can be coupled to actin cytoskeleton organisation by both MKL-SRF and YAP/TAZ-TEAD transcriptional complexes. Genome wide analysis of TEAD transcription factor binding sites reveals that TEAD cooperates with other growth factor induced transcription factors to enhance the expression of cell cycle genes. For example, ChIP-seq analysis identifies extensive overlap in the genomic binding sites of TEAD with Myc and AP1 transcription factors [219,220], suggesting that these transcriptional complexes represent a site of integration of cAMP-induced actin remodelling and growth factor signalling.

## 8. The Role of CREB

Cyclic-AMP response element binding protein (CREB), a member of the leucine zipper family of transcription factors, was the first transcription factor shown to mediate gene expression in response to elevated cAMP [221]. In 1986, Marc Montminy and Richard Goodman described a cyclic-AMP response element (CRE) in the promoter region of the somatostatin gene that was essential for expression in response to cAMP [221]. Shortly after, they reported the identification of CREB as the transcription factor that bound to the CRE to mediate cAMP induction of the somatostatin gene [221]. Transcriptional activity of CREB was then shown to be dependent on PKA-mediated phosphorylation at serine-133 [222]. The CREB family of transcription factors consists of three members, CREB, CRE modulator (CREM) and activating transcription factor-1 (ATF-1) that can all mediate transcription via CREs. Each member contains a basic region/leucine zipper (bZIP) domain that allows homo- or hetero-dimerization with other bZIP family members [223]. CREB family members also contain a-kinase-inducible domain, so named because it contains a cluster of phosphorylation sites including the regulatory serine-133 residue [224]. Phosphorylation of this serine enhances CREB-mediated transcription by promoting association of CREB with a co-activator called CREB binding protein (CBP) [224,225,226].

**Figure 3:**
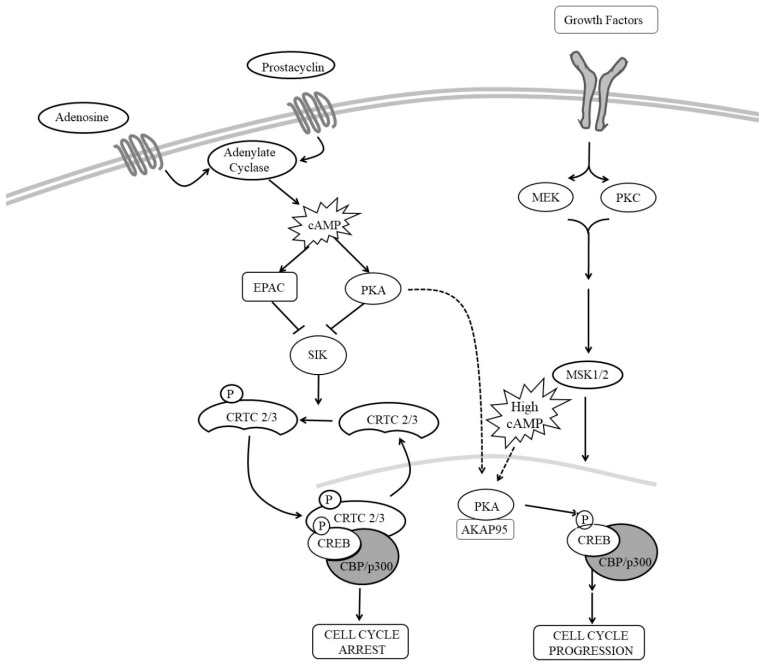
The Role of CREB in the regulation of VSMC proliferation Elevated levels of cAMP activate Protein Kinase A (PKA) and Exchange Protein Activated by cAMP (EPAC), which synergise to increase Cyclic-AMP Response Element Binding Protein (CREB)-dependent gene expression. High levels of cAMP increase CREB phosphorylation at serine-133, although low physiological levels of cAMP can induce CREB activity without increasing phosphorylation at serine-133. Cyclic-AMP stimulates nuclear translocation of the CREB regulated transcription coactivators CRTC2 and CRTC-3, which bind and activate CREB. CRTC-mediated CREB activation represses VSMC proliferation. CREB activity can also be increased in response to mitogen stimulation. This is associated with increased phosphorylation at serine-133 that can be mediated by MSK1/2 kinase, but not nuclear translocation of CRTC proteins. Activation of CREB in this way is required for maximal proliferation. Hence CREB can promote or inhibit proliferation depending on the mode of activation.

Several studies suggested that CREB contributes towards the anti-mitogenic effects of cAMP in VSMC (see Figure 3). For example, stimuli that elevate cAMP and inhibit VSMC proliferation are associated with increased CREB activity [47,91,205,227]. Inhibition of CREB using siRNA-mediated silencing or expression of dominant-negative CREB increases VSMC proliferation [228,229,230], whereas expression of constitutively active CREB mutants is inhibitory [47,228,230]. Furthermore, levels of CREB are reduced in response to a range of mitogenic stimuli and cardiovascular risk factors suggesting that CREB may play an important role in the maintenance of a normal quiescent VSMC phenotype [231]. For example, CREB levels are reduced in response to hypertension, aging, hyperlipidaemia [231] and diabetes [232] and correlate with the proliferative capacity of VSMC, being high in differentiated VSMC but reduced in phenotypically modulated cells [228]. These observations are consistent with a model where PKA-mediated phosphorylation of CREB in response to cAMP signalling inhibits VSMC proliferation. Loss of CREB in response to cardiovascular risk factors would remove this brake on proliferation and promote vascular remodelling [231]. However, many reports contradict this growth inhibitory role of CREB. Stimulation of VSMC with mitogens, including angiotensin II [233] or thrombin [234] or PDGF_BB_ [205], activates CREB; and proliferation induced by these mitogens is CREB-dependent. This suggest that CREB may be able inhibit or promote proliferation depending on the activating stimulus or mechanism of CREB activation. Although phosphorylation at serine-133 is an important step in CREB activation, it is not always necessary or sufficient for CREB-dependent gene induction. CREB can be activated independently of serine-133 phosphorylation; and not all stimuli that induce serine-133 phosphorylation induce CREB activity. For example, expression of *CREB* target genes in the mouse hippocampus in response to fear conditioning is unaffected in transgenic mice expressing CREB with a mutated serine-133 residue (CREB S133A). Furthermore, using mouse embryonic fibroblasts from CREB S133A mice, Navqi et al. demonstrated that the requirement for serine-133 phosphorylation for the forskolin induction of CREB target genes was promoter specific [235]. On the other hand, T-cell receptor stimulation of Jurkat cells induces high levels of CREB serine-133 phosphorylation without inducing target gene expression. Also, induction of CREB activity by voltage-sensitive Ca^2+^ channels is markedly reduced in a PKA-deficient PC12 cells, despite robust CREB phosphorylation at serine-133. With respect to cAMP signalling, many reports demonstrated increased phosphorylation of CREB at serine-133 in response to cAMP elevation [222,235]. However, it should be noted that almost all of these studies induce CREB activity and phosphorylation with high concentrations (10–25 µM) of the adenylyl cyclase activator forskolin or high concentrations (>200 µM) of synthetic cAMP analogues, which induce supra-physiological levels of intracellular cAMP in VSMC [25]. Importantly, stimulation of VSMC with low concentrations of forskolin or physiological GPCR agonists induces robust activation of CREB without changing phosphorylation at serine-133 [205]. Instead, these physiological cAMP stimuli activate CREB by inducing nuclear translocation of two CREB co-factors, namely CREB Regulated Transcription Coactivators-2 and -3 (CRTC2 and CRTC3). The first two members of the CRTC family, consisting of CRTC1, CRTC2 and CRTC3, were identified in a genome screen for modulators of CREB activity [236]. Under basal conditions, CRTCs are sequestered in the cytoplasm through phosphorylation dependent interactions with 14-3-3 proteins [31]. In response to elevated cAMP or calcium signalling, CRTC dephosphorylation allows their translocation into the nucleus where they bind and activate CREB in a serine-133 independent mechanism [31]. In VSMC, physiological levels of cAMP, which do not increase CREB serine-133 phosphorylation, induce nuclear translocation of CRTC2 and CRTC3. This involves both PKA and EPAC, which cooperate to induce nuclear translocation of these CRTCs and activation of CREB [205]. How EPAC promotes CRTC activation is unknown but may involve activation of Ca2+ sensitive protein phosphatase 2B (calcineurin) [237], which promotes CRTC dephosphorylation and nuclear translocation [238]. Importantly, CRTC2 and CRTC3 are both essential and sufficient for cAMP-induced activation of CREB and inhibition of proliferation. This suggests that physiological cAMP-induced CREB activity is mediated by nuclear translocation of CRTC2 and CRTC3 and occurs independently of changes in CREB serine 133 phosphorylation. In contrast, activation of CREB in response to VSMC mitogens, such as PDGF_BB,_ is associated with an increase in CREB serine-133 phosphorylation and occurs independently of CRTC nuclear translocation [205]. Importantly, CREB activity is essential for both the anti-mitogenic and mitogenic effects of these stimuli [205]. Taken together, it appears that different modes of CREB activation permit signal discrimination and allow CREB to regulate different subsets of target genes that in turn control diverse cellular responses.

## 9. Future Pharmacological and Clinical Implications

It is only after three decades of research that we are beginning to understand the complex cAMP signalling mechanisms that control VSMC proliferation. The focus now should be translating this knowledge into useful clinical therapies. This detailed knowledge has identified several potential new drug targets that may allow the vascular protective properties of cAMP to be harnessed therapeutically, while avoiding the unwanted or off target effects associated with directly targeting cAMP levels. The RhoA-ROCK pathway and remodelling of the actin cytoskeleton undoubtedly plays a central role in mediating the growth inhibitory effects of cAMP in VSMC and represents an attractive drug target. ROCK inhibitors such as Fasudil and Y27632 are available and were shown to inhibit VSMC proliferation in vitro and limit arterial injury-induced intima formation in vivo [239], while permitting endothelial regrowth [240]. This cell type specific inhibition of proliferation is an advantage over current generations of anti-mitotics used in drug eluting stents.

Other potential drug targets include the transcriptional co-factors that mediate the anti-mitogenic effects of cAMP, including MKL1/2, YAP/TAZ and the CRTCs. Although transcription factor complexes are often believed to be challenging drug targets, recent research suggest that these transcription factor:co-factor complexes may be amenable to pharmacological manipulation. Small molecule inhibitors (e.g., CCG1423) are available that block nuclear translocation of MKL1/2 into the nucleus [241], mimicking the effects of cAMP in VSMC [25]. These inhibitors reduce MKL-SRF-dependent gene expression, VSMC proliferation and injury-induced intima formation in vivo [202]. Several small molecules have also been described that disrupt interaction of the YAP and TAZ co-factors with TEAD transcription factors [242,243,244,245,246,247] that also inhibit VSMC proliferation.

## 10. Concluding Remarks

We are beginning to understand that remodelling of the actin cytoskeleton plays a central role in the complex cAMP signalling mechanisms that control VSMC proliferation. Indeed, actin cytoskeleton organisation is promoted by a diverse set of signals from the local microenvironment, including growth factors, inflammatory cytokines, the composition and mechanical properties of the ECM, and mitogenic GPCR ligands. The convergence of these signals at the cytoskeleton allows cells to generate appropriate cellular responses to multiple cues from the local environment. Moreover, through actin de-polymerisation, cAMP antagonises cellular responses to mitogenic stimuli, which is important for the maintenance of VSMC quiescence in healthy vessels and controlling resolution of repair processes following vascular insult or injury. Conversely cytoplasmic to nuclear translocation of transcriptional regulators by mitogenic factors and cAMP is a key mechanism by which signals from the plasma membrane are rapidly transduced into the nucleus. Further research into these mechanisms and the way they differ between different cell types, e.g., VSMC and endothelial cells, should lead to valuable pharmacological targets for vascular and other pathologies.
